# Metachronous carcinoma of the larynx following successful treatment of carcinoma of the bronchus.

**DOI:** 10.1038/bjc.1969.87

**Published:** 1969-12

**Authors:** R. J. Lavelle


### METACHRONOUS CARCINOMA OF THE LARYNX FOLLOWING SUCCESSFUL TREATMENT OF CARCINOMA OF THE BRONCHUS

R. J. LAVELLE

*From the Head and Neck Unit, Royal Marsden Hospital, Fulham Road, London S.W.3.*

Received for publication July 10, 1969

FOR many years now the occurrence of more than one primary malignant neoplasm in the same patient has been recognised too frequently to evoke surprise. In 1944 Warren and Ehrenreich reported the results of 2829 autopsies performed on patients who died from carcinoma; of these 194 had more than one primary tumour; on these results they calculated that a patient with one carcinoma was 11 times more likely to develop a second primary than the general population. They also found an average time interval of 3·2 years between the two tumours being recognised. The secondary primary may occur in the same or a different organ system. This series, although giving an indication of the size of the problems involved, unfortunately includes cases of asymptomatic prostatic carcinoma in middle-aged and elderly men which occurs in a high proportion of "non-cancer" patients dying of unconnected causes.

However, Epstein, Payne and Shaw (1960) conducted a statistical comparison between 867 patients with a malignant neoplasm of the air or upper food passages with 792 cases with a neoplasm of the skin or cervix uteri. Their figures showed indisputably that the presence of one neoplasm in the air and upper food passages implied an enhanced liability to the occurrence of a further malignancy in the same system.

Warren and Gates (1932) analysed 1259 cases with multicentric carcinoma; of these 120 had a primary carcinoma of the bronchus but none was associated with carcinoma of the larynx. Cahan and Montemayoer (1962) reported 240 patients seen between 1934 and 1961 at the Memorial Hospital, New York, with one primary in the bronchus and another elsewhere; in this series 60 had a second primary in the larynx of which 18 were synchronous and in 42 the lung lesion presented more than six months later. In this series there were also 3 cases of laryngeal carcinoma succeeding a bronchogenic carcinoma by more than six months; all the patients reported here were male with a long history of smoking.

Among 683 patients with established carcinoma of the larynx Frazell and Gerold (1960) found 6 with a second primary in the bronchus, but do not report any with the bronchogenic primary presenting first. Perez *et al.* (1961) reported 11 cases with "intrinsic" carcinoma of the larynx seen between 1939 and 1958 and associated with carcinoma of the bronchus; all the cases were male smokers and in 6 the tumours were metachronous (larynx first) and 5 synchronous. In 1950 Cahan *et al.* reported 1493 cases of carcinoma of the lung seen over 24 years at the Memorial Hospital, New York; they found 25 (1·6%) had a second primary elsewhere of which 4 were in the larynx.

From the various series quoted above it can be seen that the association between bronchogenic and laryngeal carcinoma is uncommon but it is still relatively more common than other associations. Before 1968 the literature contains only 154 cases of dual primary carcinoma of the larynx and lung and of these in a mere 11 was the bronchogenic lesion the first to present. The authors, and the number of cases of this latter type, are listed in Table I.

TABLE I

| Year | Author | No. of patients |
|---|---|---|
| 1955 . | Cahan . | 1 |
| 1961 . | Hollinger *et al.* . | 3 |
| 1962 . | Cahan and Montemayoer | 2 |
| 1963 . | Goorwitch . | 1 |
| 1966 . | Titche . | 1 |
| 1967 . | Sherman *et al.* . | 3 |

Before 1955 no cases of double primary neoplasms of lung and larynx in which the lung tumour preceded the laryngeal had been reported. Perusal of the case notes of all the patients treated at the Royal Marsden Hospital between 1933 and 1968 for carcinoma of the larynx failed to reveal any before 1960 who had had a previously treated carcinoma of the bronchus. However, since 1960 there have been 11 cases of carcinoma of the larynx with a past history of pneumonectomy or lobectomy for bronchogenic carcinoma. Details of these cases are shown in Table II.

It has become accepted that a diagnosis of synchronous carcinoma be made where there is less than a six months interval between the two tumours being recognised and a diagnosis of metachronous carcinoma is made when the time interval is greater than six months. For inclusion in this series as a metachronous lesion more stringent conditions have been applied: (a) the lung tumour must have been treated at least one year preceding the recognition of the laryngeal lesion; (b) there must be no evidence of recurrence of the lung lesion on chest X-ray or general clinical grounds. Any patient who satisfies these two conditions can indisputably qualify for admission to this series and cannot be confused with a metastasis from the bronchus. There are, in fact, only 13 cases recorded in the literature of metastasis to the larynx; none is from the lung, the majority being melanoma or breast or renal carcinoma. As the vast majority of laryngeal tumours are squamous cell carcinomas, as also are 40% to 50% of bronchial carcinomas, it is not, of course, possible to decide on purely histological grounds whether there are two separate primaries or whether one is a metastasis from the other.

The degree of differentiation of the tumours is also of limited value as it is clearly established that this may vary widely in different parts of the same lesion.

### DISCUSSION

During the years 1933–66 1764 patients have been treated at the Royal Marsden Hospital for carcinoma of the larynx; of these 802 (or 45%) were seen for the first time in the 7 year period 1960–66 inclusive. In this 7-year period cases 1-9 of those reported here were first seen, but of the 962 cases seen in the years 1933–59 not one had had a previously successfully treated carcinoma of the bronchus. Cases 10–11 were seen in 1967 and 1968. Although 11 cases are too few to justify any statistical judgements it does appear that since 1960 approximately 1% of the new patients attending the Royal Marsden Hospital with carcinoma of the larynx have a past history of carcinoma of the lung.

### TABLE II

| | First primary (bronchus) | | | | Second primary (larynx) | | | | | | |
|---|---|---|---|---|---|---|---|---|---|---|---|
| Case | Age | Histology | Treatment | Time interval in years | Age | Presenting symptoms | Site of origin | Histology | Treatment | Classification | Survival after treatment |
| 1 | 66 | Anaplastic | R upper lobectomy | 4 | 70 | Weakness of voice for 7 years | Supraglottic larynx | Well differentiated S.C.C. | Radiotherapy | T4 N0 M0 | 1 month |
| 2 | 62 | ? | L pneumonectomy | 6 | 68 | Haemoptysis for 1 years Increasing hoarseness for 3 years | Subglottis | Moderately differentiated S.C.C. | Radiotherapy | T3 N0 M0 | 2 months |
| 3 | 59 | Anaplastic | R upper lobectomy | 2 | 61 | Sore throat. R otalgia for 5 months | Epiglottis | Well differentiated S.C.C. | Radiotherapy | T2 N0 M0 | 6 years—still alive and well |
| 4 | 55 | Well differentiated S.C.C. | R pneumonectomy | 5 | 60 | Hoarseness. Discomfort on swallowing radiating to L ear for 4 months | Supraglottic larynx | Infiltrating S.C.C. | Radiotherapy + pharyngo-laryngectomy + block dissection | T4 N0 M0 | 4 years—died of carotid erosion |
| 5 | 48 | ? | L pneumonectomy | 16 | 64 | 6 months hoarseness | Vocal cord | Carcinoma *in situ* | Radiotherapy | T1 N0 M0 | 3rd primary in tongue but alive and well 4 years later |
| 6 | 60 | Undifferentiated polygonal cell carcinoma | L lower lobectomy | 3 | 63 | Hoarseness for 4 months | Vocal cord | Well differentiated S.C.C. | Radiotherapy followed 2 years later by laryngectomy | T2 N0 M0 | Alive and well 2 years since laryngectomy |
| 7 | 40 | ? | L pneumonectomy | 13 | 53 | Hoarseness for 2 months | Vocal cord | Moderately differentiated S.C.C. | Radiotherapy | T1 N0 M0 | No recurrence in larynx but further Ca. R lung and died 2 years after radiotherapy |
| 8 | 68 | Poorly differentiated S.C.C. | Right lower and middle lobectomy | 4 | 72 | Hoarseness for 4 months | Vocal cord | Well differentiated S.C.C. | Radiotherapy | T1 N0 M0 | Alive and well after 3 years |
| 9 | 68 | Poorly differentiated S.C.C. | L upper lobectomy | 3 | 71 | Sore throat R otalgia on swallowing for 6 months | Supraglottic | Moderately differentiated S.C.C. | Radiotherapy | T4 N0 M0 | 3 years. Died of further Ca. lung with no evidence of laryngeal recurrence |
| 10 | 52 | S.C.C. | L pneumonectomy | 17 | 69 | Hoarseness for 5 months | Supraglottic | Moderately differentiated S.C.C. | Radiotherapy | T3 N0 M0 | 3 months |
| 11 | 69 | S.C.C. | R pneumonectomy | 4 | 73 | Hoarseness. Haemoptysis pain on swallowing liquids for 8 months | Vocal cord | Poorly differentiated S.C.C. | Radiotherapy | T3 N0 M0 | 2 months |

S.C.C. Squamous cell carcinoma.

It is difficult to answer why none should have been seen in the 27 years between 1933 and 1960 and 11 between 1960 and 1968. The increased frequency of carcinoma of the bronchus increases the potential pool of patients and the improved results in treatment of this disease has meant more patients are surviving a greater length of time. Acceptance of these two premises suggests that metachronous carcinoma of the larynx following successful pneumonectomy or lobectomy will become increasingly common. This being so, the thoracic surgeon or radiotherapist following up successfully treated cases of carcinoma of the bronchus will see more often subsequent cases of laryngeal carcinoma.

Unfortunately the presenting symptom of carcinoma of the larynx may in every case be regarded as evidence of recurrence of the initial bronchial disease; this will in fact be true in many patients but if no evidence of intra-thoracic recurrence is found examination of the larynx by indirect laryngoscopy is essential. If this is not unimpeachably normal further investigation by tomography and, if necessary, direct laryngoscopy should be performed.

Most revealing in this respect is case 2. Here the patient five years after pneumonectomy developed haemoptysis. Not unnaturally a pulmonary recurrence was suspected but three bronchoscopies and a year passed before the larynx was examined, by which time a $T_3$ tumour with a fixed hemilarynx was present. Any patient developing hoarseness, weakness of the voice, haemoptysis, dysphagia, discomfort in the throat, a palpable mass in the neck or otalgia following apparently successful treatment of a bronchogenic carcinoma should automatically be subjected to full laryngological investigation.

A past history of carcinoma of the bronchus should not affect the management of a subsequent carcinoma of the larynx. Inevitably as more double primaries are controlled more third and fourth primaries will appear. Again although 11 cases are too few to draw any definite statistical conclusions, it is worthy of comment that both cases 5 and 7 developed third primaries of which that in case 5 has also been controlled; he is now a man of 68 years in apparently good health, but it must be considered very likely that he will in the upper food or air passages develop a fourth primary. The other third primary, case 7, unfortunately appeared in his only remaining lung and was not amenable to radical treatment.

### SUMMARY

(a) The increasing frequency of carcinoma of the larynx following successful treatment of carcinoma of the bronchus is indicated.

(b) Although no definite explanation can at the present time be advanced, the improved results in treating carcinoma of the bronchus which is itself becoming commoner partially explains why subsequent carcinoma of the larynx is seen more commonly.

(c) Eleven cases are reported increasing the total recorded to 22.

(d) As in previous series all the cases here are male. The importance of full laryngological investigation following successful treatment of bronchial carcinomas is emphasised when symptoms which may indicate either pulmonary recurrence or a further laryngeal primary occur.

(e) Although it was not possible to ascertain with certainty the smoking habit of all these patients there were no definite non-smokers among them.

I should like to express my thanks to Mr. H. J. Shaw, Dr. M. Lederman and Dr. V. Dalley for allowing me to use their patients for publication in this article.

### REFERENCES

CAHAN, W. G.—(1955) *Am. J. Surg.*, **89**, 494.
CAHAN, W. G., BUTLER, F. S., WATSON, W. L. AND PAUL, J. L.—(1950) *J. thorac. Surg.*, **20**, 335.
CAHAN, W. G. AND MONTEMAYOER, P. B.—(1962) *J. thorac. cardiovasc. Surg.*, **44**, 309.
EPSTEIN, S. S., PAYNE, P. M. AND SHAW, H. J.—(1960) *Cancer N.Y.*, **13**, 137.
FRAZELL, E. I. AND GEROLD, F. B.—(1960) *Post-grad. Med.*, **27**, 394.
GOORWITCH, J.—(1963) *J. Am. med. Ass.*, **183**, 375.
HOLLINGER, P. H., JOHNSTON, K. C., JENSIK, R. J. AND HURZELER, D.—(1961) *Ann. Otol. Rhinol. Lar.*, **70**, 475.
PEREZ, P. E., BERNATZ, P. E., DEVINE, K. D. AND WOONER, L. B.—(1961) *J. Am. med. Ass.*, **177**, 596.
SHERMAN, J. O., STALEY, G. J. AND SHIELDS, T. W.—(1967) *Archs Surg.*, **94**, 550.
TITCHE, L. I.—(1966) *Archs Otolar.*, **83**, 598.
WARREN, S. AND EHRENREICH, T.—(1944) *Cancer Res.*, **4**, 554.
WARREN, S. AND GATES, O.—(1932) *Am. J. Cancer*, **16**, 1359.


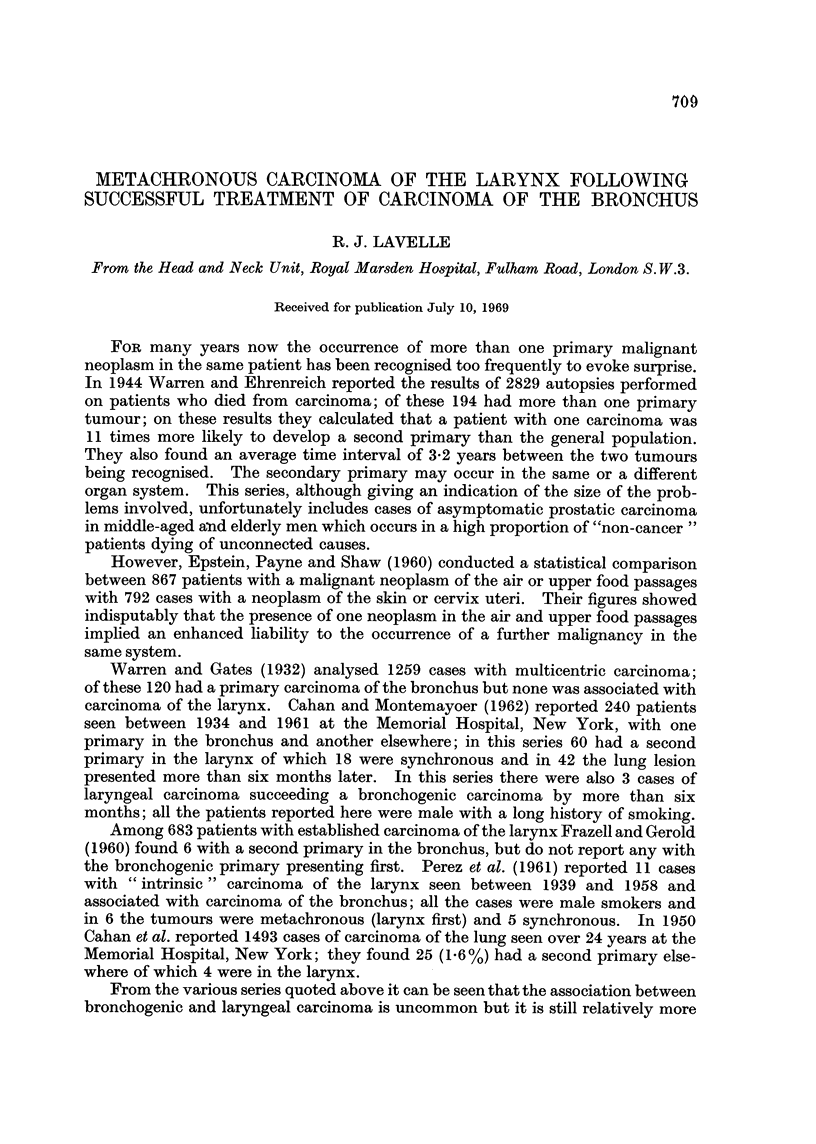

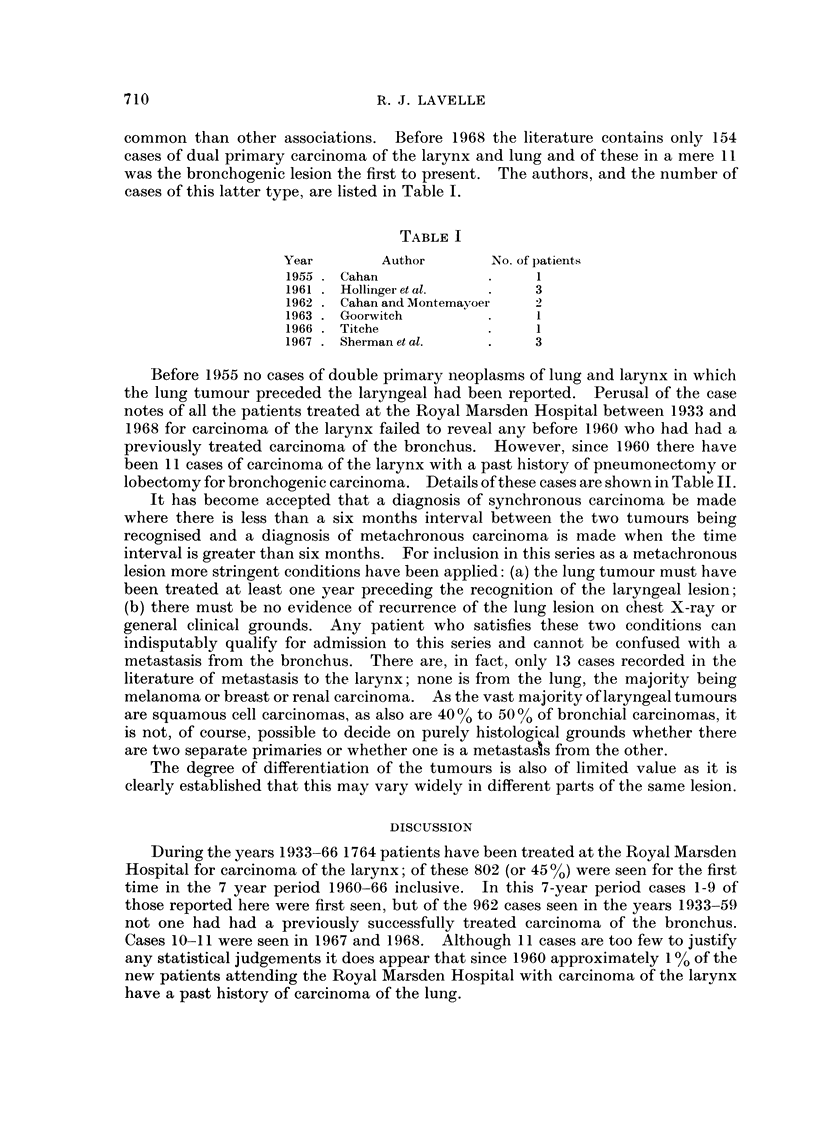

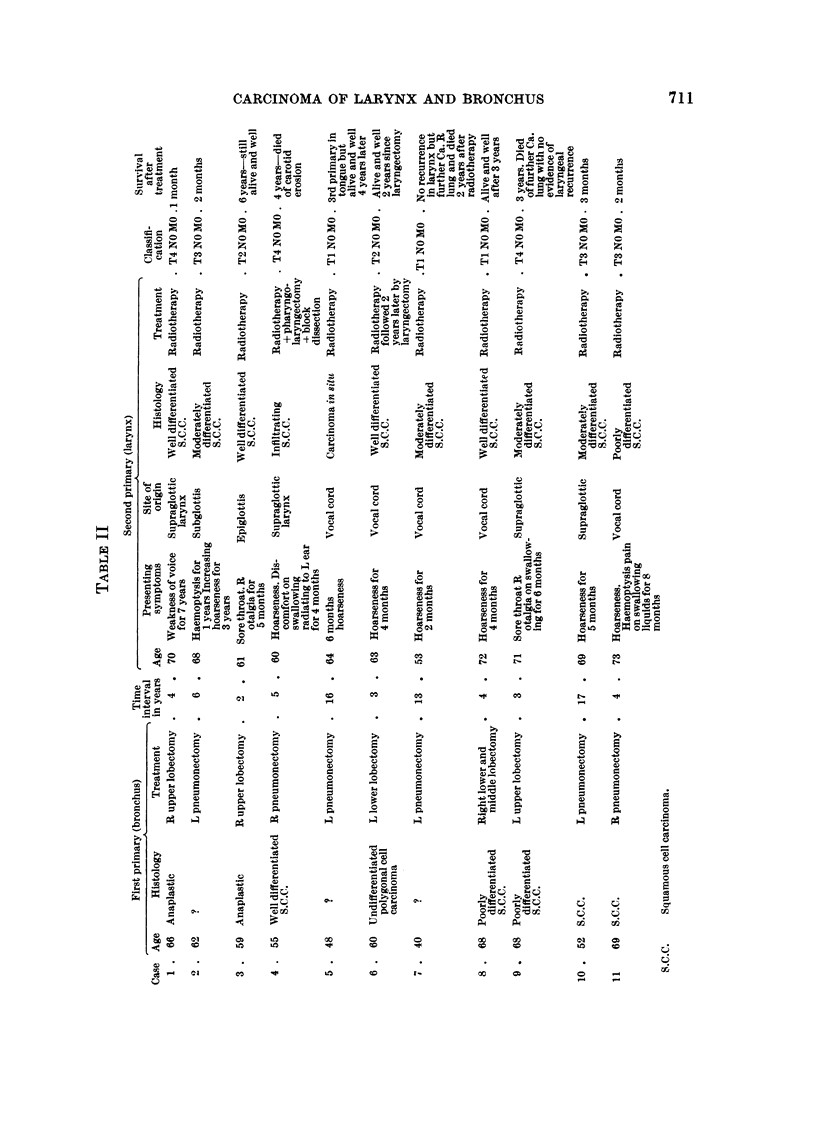

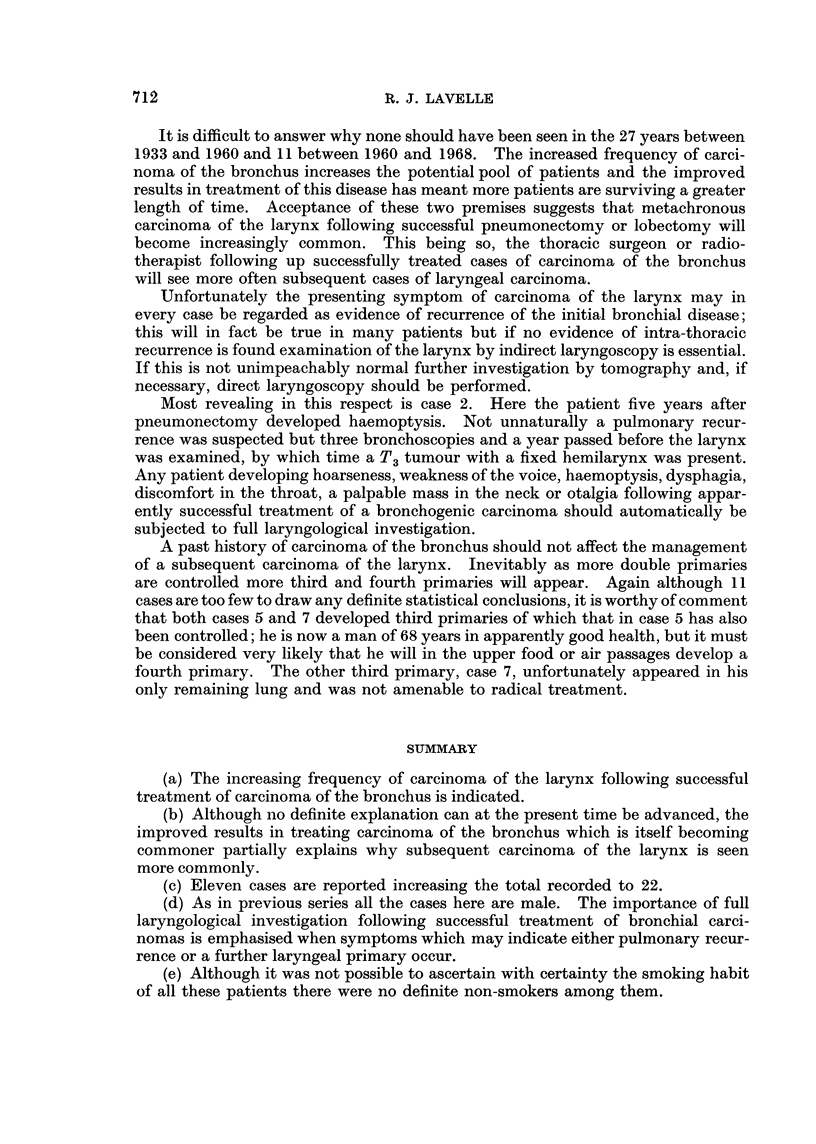

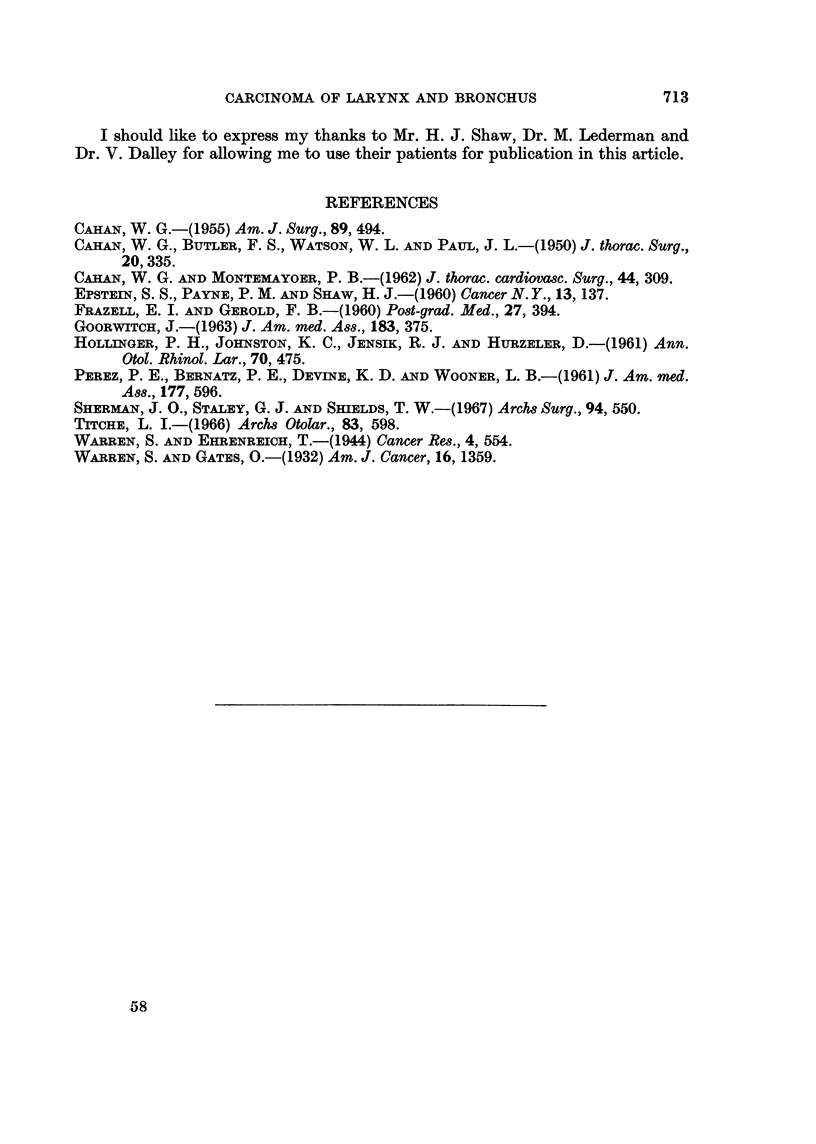


## References

[OCR_00404] CAHAN W. G., BUTLER F. S., WATSON W. L., POOL J. L. (1950). Multiple cancers: primary in the lung and other sites.. J Thorac Surg.

[OCR_00406] CAHAN W. G., MONTEMAYOR P. B. (1962). Cancer of the larynx and lung in the same patient. A report of 60 cases.. J Thorac Cardiovasc Surg.

[OCR_00409] GOORWITCH J. (1963). Consecutive primary carcinomas of bronchus and larynx.. JAMA.

[OCR_00411] HOLINGER P. H., JOHNSTON K. C., JENSIK R. J., HURZELER D. (1961). Laryngeal and bronchial cancer. A study of double primary malignancy.. Ann Otol Rhinol Laryngol.

[OCR_00415] PEREZ P. E., BERNATZ P. E., DEVINE K. D., WOOLNER L. B. (1961). Associated primary endolaryngeal carcinoma and bronchogenic carcinoma.. JAMA.

[OCR_00419] Sherman J. O., Staley C. J., Shields T. W. (1967). Double primary tumors of the larynx and lung.. Arch Surg.

[OCR_00420] Titche L. L. (1966). Carcinoma of the larynx following carcinoma of the lung.. Arch Otolaryngol.

